# A Novel Carrier Loop Algorithm Based on Maximum Likelihood Estimation (MLE) and Kalman Filter (KF) for Weak TC-OFDM Signals

**DOI:** 10.3390/s18072256

**Published:** 2018-07-13

**Authors:** Wen Liu, Xinmei Bian, Zhongliang Deng, Jun Mo, Buyun Jia

**Affiliations:** School of Electronic Engineering, Beijing University of Posts & Telecommunications, No. 10 Xitucheng Road, Haidian District, Beijing 100876, China; bianxinmei@bupt.edu.cn (X.B.); dengzhl@bupt.edu.cn (Z.D.); mojun@bupt.edu.cn (J.M.); jiabuyun@bupt.edu.cn (B.J.)

**Keywords:** carrier loop, MLE, LM, KF, TC-OFDM

## Abstract

Digital broadcasting signals represent a promising positioning signal for indoors applications. A novel positioning technology named Time & Code Division-Orthogonal Frequency Division Multiplexing (TC-OFDM) is mainly discussed in this paper, which is based on China mobile multimedia broadcasting (CMMB). Signal strength is an important factor that affects the carrier loop performance of the TC-OFDM receiver. In the case of weak TC-OFDM signals, the current carrier loop algorithm has large residual carrier errors, which limit the tracking sensitivity of the existing carrier loop in complex indoor environments. This paper proposes a novel carrier loop algorithm based on Maximum Likelihood Estimation (MLE) and Kalman Filter (KF) to solve the above problem. The discriminator of the current carrier loop is replaced by the MLE discriminator function in the proposed algorithm. The Levenberg-Marquardt (LM) algorithm is utilized to obtain the MLE cost function consisting of signal amplitude, residual carrier frequency and carrier phase, and the MLE discriminator function is derived from the corresponding MLE cost function. The KF is used to smooth the MLE discriminator function results, which takes the carrier phase estimation, the angular frequency estimation and the angular frequency rate as the state vector. Theoretical analysis and simulation results show that the proposed algorithm can improve the tracking sensitivity of the TC-OFDM receiver by taking full advantage of the characteristics of the carrier loop parameters. Compared with the current carrier loop algorithms, the tracking sensitivity is effectively improved by 2–4 dB, and the better performance of the proposed algorithm is verified in the real environment.

## 1. Introduction

With the development of mobile Internet and mobile smart devices, location-based services have become the focus in many wireless network applications. In open outdoor areas, Global Navigation Satellite Systems (GNSS) can provide precise location information for outdoor activities, however, in buildings, urban canyons, and forested areas, the effectiveness of positioning using GNSS is limited, making it impossible to provide high-precision location-based services. Typical studies on the above problems in such harsh environments can be found in [1,2,3,4,5]. A general framework to characterize the localization accuracy by unifying localization information of wideband wireless networks is proposed in [1]. Reference [2] established the fundamental limits of wideband cooperative location-aware networks. Centimeter accuracy indoor localization in an Assisted Living (AL) system is achieved for a 5G systems using millimeter-wave signals, which can be found in [3]. Reference [4] proposed a range information model which is a function of wireless environment, signal features and energy detection techniques. Soft range information-based localization can be found in [5] for high accuracy network localization. Meanwhile, precise localization is of great importance in many applications such as vehicular networks [6]. A state of the art vehicular communication system for the cooperative awareness of connected vehicles is proposed in [7].

Studies show that people spend more than 80% of their time in indoors environments [8]. With the increasing number of tall buildings, indoor location-based services have emerging applications in commercial applications and public safety. As the main positioning technology, GNSS has many significant advantages outdoors, such as a large coverage area, high positioning accuracy, and superior navigation performance [9]. However, buildings obstruct, reflect, and diffract the GNSS signals, the positioning performance is limited in indoor environments and cities. Recently, terrestrial radio positioning systems and their enhancements to the GNSS have drawn increasing attention, and the digital broadcasting signal is a promising positioning signal for indoors uses [10]. As for DVB-T/H signals, a different low-complexity solution to perform the coupling channel estimation in an on-channel repeater can be found in [11,12,13]. This paper mainly studies a novel terrestrial radio positioning system called Time & Code Division-Orthogonal Frequency Division Multiplexing (TC-OFDM), which is based on the China Mobile Multimedia Broadcasting (CMMB) system. The TC-OFDM system multiplexes the CMMB signal and pseudorange noise (PRN) codes in the same frequency band, and the positioning performance can be achieved by adding some simple modifications to the deployed CMMB facilities. The positioning part of the TC-OFDM system is a direct-sequence spread spectrum code division multiple access (DSSS-CDMA) system employing binary phase shift keying (BPSK) modulation, and [10] describes the TC-OFDM system in detail. Compared with the GNSS, the TC-OFDM system has many potential advantages: the TC-OFDM signal transmission power is stronger, the frequency band is the U band. The TC-OFDM receiver demodulates PRN codes for high accuracy positioning, which can overcome the positioning limitations caused by the CMMB single network coverage.

Weak signal strength is the main factor that limits the performance of the receiver tracking loop to improve tracking sensitivity. Meanwhile, the indoor environment is more complex than outdoors and the signal strength is significantly attenuated, which restricts the tracking performance of the existing receivers for weak TC-OFDM signals. The main reason is that the accuracy of the residual carrier estimation by the existing carrier loop algorithm is not ideal under weak signal conditions. Since the carrier loop is the weakest link in the receiver tracking loop, the superior carrier loop performance can improve the tracking sensitivity of the receiver for weak TC-OFDM signals to a certain degree [14]. The carrier loop is usually divided into a frequency-locked loop (FLL) and phase-locked loop (PLL) [15,16]. The FLL has good dynamic performance, but the carrier phase measurement accuracy is lower due to the wide noise bandwidth [17]. The PLL tracks the received signal more closely and has the high carrier phase measurement accuracy, but it has poor tolerance of dynamic stress [18]. Due to the complexity of the indoor environment, the received signal strength is greatly attenuated. Therefore, the existing carrier loop performance needs to be improved for better performance in indoor weak signal environment. Currently, the methods to improve the carrier loop performance are divided into three categories: the KF-based PLL [19], the algorithms based on the Maximum likelihood estimation (MLE) algorithm [20], and the FLL-assisted PLL [21]. The KF-based PLL includes Extended Kalman Filter (EKF) and Unscented Kalman Filter (UKF) algorithms [22,23]. The observation model in the carrier loop is nonlinear, which means the observed signal vector and the estimated state vector are nonlinear in the carrier loop. The EKF and UKF are utilized to expand the nonlinear observation functions using Taylor series and keep the linear part of the function while ignoring the higher order nonlinear part. However, the EKF and UKF based on linear minimum mean square error estimation criterion represent a sub-optimal carrier loop scheme, which still produces a large parameter estimation error for weak received signals. Methods based on FLL-assisted PLL can ensure the dynamic of the carrier loop but the estimation accuracy is limited in a weak signal environment [24]. Reference [25] uses the MLE to adjust the Doppler frequency in a high dynamics receiver carrier loop, but it does not make a detailed analysis for weak received signals. Iterative and non-iterative MLE are proposed to estimate the signal Doppler frequency and code phase in [26,27]. These two algorithms need to deal with real-time IF data, which means a heavy calculation burden.

This paper proposes a novel carrier loop algorithm based on MLE and KF for weak TC-OFDM signals. The main idea is to combine the MLE with KF to effectively estimate the carrier loop parameters and smooth the estimation results, so as to obtain an accurate estimation of the residual carrier for weak TC-OFDM signals and improve the receiver tracking sensitivity indoors. With the novel algorithm, the MLE discriminator function replaces the current carrier loop discriminator. The Levenberg-Marquardt (LM) algorithm is utilized to obtain the MLE cost function consisting of signal amplitude, residual carrier frequency and carrier phase, and the MLE discriminator function is derived from the corresponding MLE cost function. The KF is used to smooth the MLE discriminator function results, which takes the carrier phase estimation, the angular frequency estimation and the angular frequency rate as the state vector.

The rest of this paper is organized as follows: Section 2 presents the signal model. The proposed carrier loop algorithm is introduced in Section 3. Section 4 presents the simulation and real data test results of the comparison between different carrier loop algorithms. Finally, Section 5 concludes the work.

## 2. Signal Model

**Figure 1:**
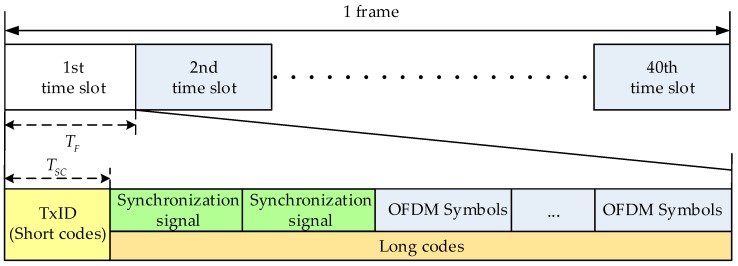
TC-OFDM signal Frame Structure.

The CMMB signal and pseudorange noise (PRN) codes are multiplexed in the same frequency band. Figure 1 shows the structure of the TC-OFDM signal. The duration of each frame of the CMMB signal is 1 s, and each frame consists of 40 time slots. The duration of each time slot is 25 ms. The CMMB system uses orthogonal frequency division multiplexing (OFDM) to transmit the broadcasting signal. Two kinds of pseudo codes are superimposed on the CMMB signals to provide high accuracy positioning services, which are named short codes and long codes, respectively. The beginning of the frame has the TxID and two synchronization signals. The TxID is empty in the practice CMMB signal, so the short codes are superimposed on the TxID to distinguish different base stations by using different pseudo codes signals. Thanks to the fact the TxID is empty, the short codes and the CMMB signals have the same transmission power. In order not to affect the normal communication of the CMMB signal, the power of long codes is lower than the CMMB signal by 20 dB [10]. In the positioning, the receiver uses short codes to achieve acquisition and long codes to achieve stable tracking.

The TC-OFDM signal of the *n*th time slot can be expressed as:

(1) $$s_{k}^{\left(i\right)}(t)=\left\{ \begin{array}{ll} s_{CMMB}(t)+c_{SC}^{\left(i\right)}(t) & (k-1)T_{F}\leq t<(k-1)T_{F}+T_{SC}\\ s_{CMMB}(t)+\alpha c_{LC}^{\left(i\right)}(t) & \;(k-1)T_{F}+T_{SC}\leq t\leq kT_{F}\\ 0 & \;others \end{array}\right.$$

where the *S_CMMB_*(*t*) is the CMMB signal, *C_SC_*(*t*) and *C_LC_*(*t*) is short codes and long codes respectively. The superscript *i* represents the base station number. *T_F_* is the time length of the time slot and *T_SC_* is the time length of the short codes. The term α is the signal attenuation factor.

In order to transmit the positioning-assisted information, the PRN codes sent by the base station are modulated with the positioning-assisted information, and each slot modulates one bit positioning-assisted information. The TC-OFDM signal of *i*th base station can be written as:

(2) $$s^{\left(i\right)}(t)=\sum_{k=-\infty }^{\infty }d^{\left(i\right)}(t)s_{k}^{\left(i\right)}(t)$$

where *d*(*t*) denotes the positioning-assisted information. The signal transmitted by the *i*th base station is:

(3) $$S^{\left(i\right)}(t)=s^{\left(i\right)}(t)\cos(2\pi f_{c}t+\varphi_{0,i}(t))$$

where *f_c_* is the carrier frequency, *φ_0,i_*(*t*) is the initial phase.

The signal from the *i*th base station is received by the receiver radio frequency (RF) antenna. The output signal of the RF antenna can be written as:

(4) $$r(t)=\;\sum_{i=1}^{N}A^{\left(i\right)}s^{\left(i\right)}(t-\tau_{i})\cos(2\pi (f_{c}+f_{d,i})t+\varphi_{0,i}(t))+\omega (t)$$

where *N* denotes the signal received from *N* different base stations. *A*^(*i*)^ is the signal amplitude, *τ_i_* is the incoming signal delay, *f_c_* is the carrier frequency, *f_d,i_* is the incoming Doppler shift, *φ_0,i_*(*t*) is the initial phase, *ω*(*t*) is the additive Gaussian white noise (AWGN) with zero mean and variance *σ_n_*^2^.

After the receiver RF front-end, down-conversion, low-pass filter and analog-digital conversion module (ADC), the digital intermediate frequency (IF) signal is expressed as:

(5) $$r_{IF}{}^{\left(i\right)}(nT_{s})=A_{IF}s^{\left(i\right)}(nT_{s}-\tau_{i})e^{j2\pi (f_{IF}+f_{d,i})nT_{s}+\varphi_{0,i}}+\omega (n)$$

where *T*_s_ is the sampling duration, *A_IF_* is the IF signal amplitude, *τ_i_* is the incoming signal delay, *f_IF_* is the IF frequency, *f_d,i_* is the Doppler shift, *φ_0,i_* is the initial carrier phase, *ω*(*n*) is the additive Gaussian white noise (AWGN) with zero mean and variance *σ_n_*^2^.

The IF signal is sent to the baseband processor for acquisition [10], tracking and data demodulation. A rough estimation of carrier frequency and code phase of the received signal is obtained from the acquisition process, which initialize the related parameters of the tracking channel. Then the estimation of these two signal parameters is refined step by step through the tracking loop. The numerically controlled oscillator (NCO) in the tracking loop produces mutually orthogonal sine and cosine signals, which can be created as:

(6) $$\{\begin{array}{c} \mu_{os}(t)=\sin(2\pi f_{NCO}t+\varphi_{NCO})\\ \mu_{oc}(t)=\cos(2\pi f_{NCO}t+\varphi_{NCO}) \end{array}$$

where $f_{NCO}$ and $\varphi_{NCO}$ is the carrier *NCO* frequency and initial phase respectively.

In this research, the TCXO is used in the TC-OFDM receiver, and the accuracy of the TCXO is 0.5 ppm. After many real tests, we find that the phase noise of the receiver clock is smaller than white Gaussian noise in the research of the TC-OFDM meter-level positioning accuracy. Also, we can use the TCXO phase noise estimation algorithm to predict the actual phase noise. However, adding the TCXO phase estimation algorithm has less influence on the proposed algorithm in this research. Therefore, the effect of phase noise can be ignored in this research. After carrier stripped, the coherent integration result of I and Q channel signals by integration and dumping module can be created as:

(7) $$\{\begin{array}{c} Corr(n)=A_{mp}s(n)\sin c(\Delta fT_{coh})e^{\left(j(2\pi \Delta fnT_{coh}+\Delta \varphi \right)}+\omega (n)\\ A_{mp}=A_{IF}(n)T_{coh},\;\Delta f=f_{d}-f_{NCO},\;\Delta \varphi =\varphi_{IF}-\varphi_{NCO} \end{array}$$

where *Corr*(*n*) represents the *n*th coherent integration result, *T_coh_* is the coherent integration duration, *s*(*n*) denotes the positioning-assisted information, *A_mp_*, Δ*f* and Δ*φ* represent the signal amplitude, carrier and phase residual respectively. In order to avoid bit transition, the coherent integration duration used generally do not exceed one time slot duration. We assume that in the coherent integration duration, *s*(*n*) can be regarded as a fixed value.

For the convenience of analysis, we assume that the local code is strictly aligned with the received signal. When in the coherent integration duration, the parameters *A_mp_*, Δ*f* and Δ*φ* have minor changes which can be regarded as unknown nonrandom parameters. In the tracking process, the term of sinc(Δ*fT_coh_*) ≈ 1 when Δ*f* is small enough. Then Equation (8) can be simplified as:

(8) $$Corr(n)\approx A_{mp}s(n)e^{\left(j(2\pi \Delta fnT_{coh}+\Delta \varphi \right)}+\omega (n)$$

**Figure 2:**
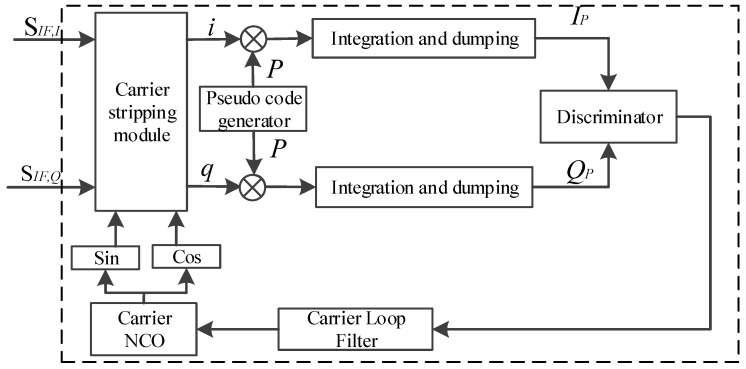
Conventional carrier loop structure.

The conventional carrier loop structure is shown in Figure 2. The coherent integration results of Equation (8) is sent into the carrier discriminator to estimate carrier frequency. Then the Carrier NCO is adjusted in real time based on the carrier frequency filtering results from the loop filter.

## 3. MLE Parameter Estimation Model

In the case of an unknown prior distribution of estimated parameters, MLE provides the parameter estimation with the smallest error variance when the parameters to be estimated is symmetric and unimodal distributions. In terms of the linear discrete-time system, linear KF is the optimal filter [28]. Therefore, the MLE combined with KF can make an accurate estimation of the parameters in the carrier loop, which can improve the tracking loop performance. The MLE is used to nonlinearly estimate the carrier loop parameters of interest, and then KF is applied to smooth the estimated parameters from MLE. Therefore, the proposed algorithm of combining MLE with KF is the optimal solution for the TC-OFDM carrier frequency accurately tracking in the weak TC-OFDM signal environment.

### 3.1. The Principle of MLE

**Figure 3:**
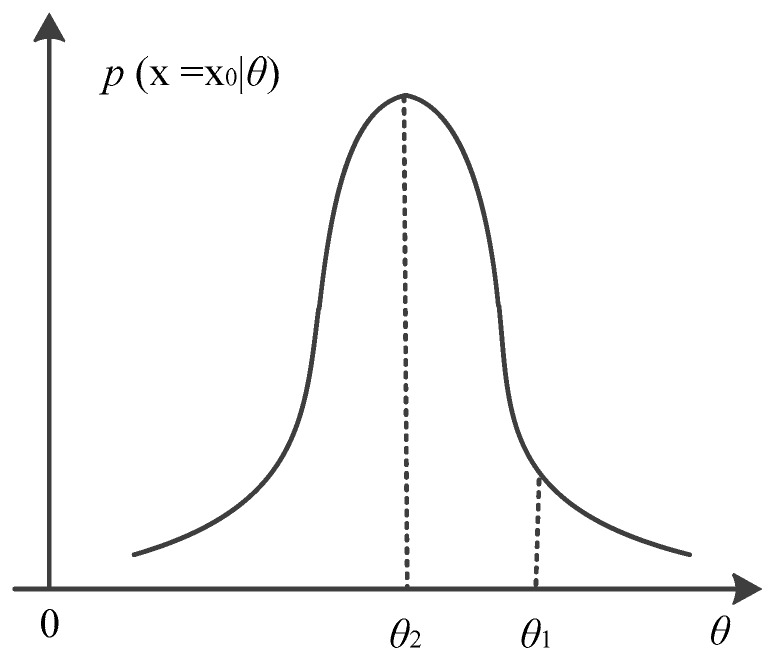
The principle of MLE.

MLE is often used to estimate unknown non-random parameters. MLE is defined as the estimator of *θ* value that maximizes the likelihood function. The estimated value is denoted as *θ_MLE_*(*x*). We take the MLE for a single-parameter *θ* as an example. For an unknown non-random estimation *θ*, the probability density function *p*(*x*|*θ*) of the observation vector (denotes as *x*) is called the likelihood function. The basic principle of MLE can be explained as follows: for a selected *θ*, MLE consider the probability *p*(*x*|*θ*)*dx* that *x* falls in a small area, and take the corresponding *θ* whose *p*(*x*|*θ*)*dx* is the largest as the estimated *θ_MLE_*(*x*). As shown in Figure 3, the likelihood function is obtained after *x* = *x*_0_, then we can draw a curve between the likelihood function and the estimated value of *θ.* The value of *p*(*x*|*θ*)*dx* for each *θ* means the probability that *x* falls within d*x*, and d*x* centered on *x_0_* in the observation space R for *θ*. When *x* = *x*_0_, it can be seen that *θ* = *θ*_1_ is unreasonable, so we choose *θ* = *θ*_2_ as the estimation, and the *θ* that maximizes *p*(*x* = *x*_0_|*θ*) is selected as the estimated value *θ_MLE_*(*x*) within a range allowed by the estimated *θ*.

### 3.2. MLE Cost Function

Given a set of observations *x*, the MLE of *θ* is the *θ_MLE_*(*x*) at which the probability density function is maximized:

(9) $$\theta_{MLE}(x)=\arg\underset{\theta }{\max}p(x|\theta )$$

According to the above analysis, when the likelihood function *p*(*x*|*θ*) is known, *θ_MLE_*(*x*) can be obtained by the following equation and the logarithmic form is given in Equation (11):

(10) $${\frac{\partial p(x|\theta )}{\partial \theta }|}_{\theta =\theta_{MLE}}=0$$

(11) $${\frac{\partial \ln p(x|\theta )}{\partial \theta }|}_{\theta =\theta_{MLE}}=0$$

Since MLE is considered a valid parameter estimation algorithm in the tracking loop of the TC-OFDM receiver, we use MLE to effectively estimate the residual carrier frequency for weak TC-OFDM signals indoors. Meanwhile, the MLE produces the minimum variance of estimation error, when the statistical distribution of the estimated signal parameters in an uncertain interval is unknown [29]. When it comes to multi-parameter problems, the MLE of signal parameters is the estimation of signal parameters when the joint conditional probability density of a set of signal observations gets maximized. Since the coherent integration time in the algorithm is 1.6 ms which is shorter than the loop update time, we assume that the carrier is correctly tracked within such a short coherent integration time. Therefore, the parameters to be estimated can be regarded as unknown constants over the interval. Meanwhile, *N* is defined as the number of consecutive coherent integration results in this paper. The coherent integration results are adopted as observation sample parameters of MLE, which contains the signal amplitude, residual carrier and phase value in Equation (8). The value of *N* is selected to ensure that the parameters to be estimated remain constant over the entire interval, and *N* is closely related to the dynamic bandwidth of the receiver. The TC-OFDM system is for indoor positioning, which is mainly under low dynamic cases. In the low dynamic case, signal amplitude is the main change parameter. We use the MLE to estimate the signal amplitude, and other parameters (including residual carrier and phase) are little changed under such the low dynamic case. Also, the simulation and real test are used to determine the value of *N* in this research. Therefore, in order to obtain the MLE of the unknown *A_mp_*, Δ*f* and Δ*φ* in Equation (8), the joint probability density function of *N* consecutive coherent integration results is obtained according to [30]:

(12) $$p(Corr_{N}|A_{mp},\Delta f,\Delta \varphi )=\frac{1}{(2\pi \sigma^{2}{)}^{N}}e^{\left(-\frac{1}{2\sigma^{2}}(Corr_{N}-{\overset{\wedge }{Corr}}_{N})W{\left(Corr_{N}-{\overset{\wedge }{Corr}}_{N}\right)}^{H}\right)}$$

where *Corr_N_* = [*Corr*(0), *Corr* (1), *……*, *Corr* (*N* − 1)] represents *N* consecutive coherent integration results, ${\overset{\wedge }{Corr}}_{N}$ is the estimated value of $Corr_{N}$. *W* is the diagonal matrix which stands for the weight factor of *Corr*(*n*), superscript *H* denotes the matrix transpose and conjugate operations.

The MLE minimum variance unbiased estimation can be achieved by obtaining the maximum value of (10) to get the values *A_mp_*, Δ*f* and Δ*φ*. The diagonal elements of the diagonal matrix *W* are set as 1 to obtain the log-likelihood cost function of Equation (12) as in [31,32]:

(13) $$\begin{array}{l} \Lambda (\mu |Corr_{N})=-\frac{1}{2\sigma^{2}}(Corr_{N}-{\overset{\wedge }{Corr}}_{N}){\left(Corr_{N}-{\overset{\wedge }{Corr}}_{N}\right)}^{H}-N\ln(2\pi \sigma^{2})\\ =-\frac{1}{2\sigma^{2}}{\left|Corr_{N}-{\overset{\wedge }{Corr}}_{N}\right|}^{2}-N\ln(2\pi \sigma^{2})\\ =-\frac{1}{2\sigma^{2}}\sum_{n=0}^{N-1}{\left(real(Corr_{n})-A_{mp}\cos(\theta )\right)}^{2}-\frac{1}{2\sigma^{2}}\sum_{n=0}^{N-1}{\left(imag(Corr_{n})-A_{mp}\sin(\theta )\right)}^{2}-N\ln(2\pi \sigma^{2})\\ =-\frac{1}{2\sigma^{2}}\sum_{n=0}^{N-1}\left\{{\left|Corr_{n}\right|}^{2}+A_{mp}{}^{2}-2A_{mp}[real(Corr_{n})\cos(\theta )+imag(Corr_{n})\sin(\theta )]\right\}-N\ln(2\pi \sigma^{2}) \end{array}$$

where *Corr_n_* = *Corr*(*n*), *Corr_N_* = [*Corr*(0), *Corr* (1), ……, *Corr* (*N* − 1)] is *N* consecutive coherent integration results. *μ* = [*A_mp_* Δ*f* Δ*φ*]*^T^* represents the signal parameters to be estimated, *n* = 0…*N* − 1, *θ* = 2*π*Δ*fnT_coh_* + Δ*φ*, real(·) and imag(·) represent the real and imaginary part respectively.

By finding the maximum value of Equation (13) to get the estimated value of *μ*, which can be expressed as:

(14) $$\frac{\partial \Lambda (\mu |Corr_{N})}{\partial \mu }=0$$

Deriving the partial derivative of *μ*:

(15) $$\frac{\partial \Lambda }{\partial A_{mp}}=-\frac{1}{\sigma^{2}}\sum_{n=0}^{N-1}\left\{A_{mp}-[real(Corr_{n})\cos(\theta )+imag(Corr_{n})\sin(\theta )\right]\}$$

when $\frac{\partial \Lambda }{\partial A_{mp}}=0$, the estimation value of *A_mp_* is obtained:

(16) $$A_{mp}=\frac{1}{N}\sum_{n=0}^{N-1}\left[real(Corr_{n})\cos(\theta )+imag(Corr_{n})\sin(\theta )\right]$$

The signal amplitude *A_mp_* estimated in the MLE is used to subsequently adjust the observation noise covariance matrix *R* in the KF. Since the items of |*Corr_n_*|^2^, *A_mp_*^2^ and *Nln*(2*πσ*^2^) in Equation (13) do not affect Λ partial derivative of Δ*f* and Δ*φ* respectively, we can remove such irrelevant items and the cost function is simplified as:

(17) $$M=\sum_{n=0}^{N-1}\left[real(Corr_{n})\cos(\theta )+imag(Corr_{n})\sin(\theta )\right]$$

where *θ* = 2*π*Δ*fnT_coh_* + Δ*φ*, real(·) and imag(·) represent the real and imaginary part respectively.

We can find that if *θ* is obtained, then *A_mp_* can be calculated by Equation (16). According to the relationship between *θ*, Δ*f* and Δ*φ*, *θ* can be obtained as long as we get the value of Δ*f* and Δ*φ*. Thus *A_mp_* can be acquired by Equation (16), the rest is to estimate the value of *θ*, which is equivalent to get the value of Δ*f* and Δ*φ*. Solving the problem of *θ* in Equation (17), which the essence is to obtain the values of Δ*f* and Δ*φ*. Getting the value of Δ*f* and Δ*φ* can be regarded as the process of solving a two-dimensional optimal problem. Therefore, the MLE solution of Δ*f* and Δ*φ* in *θ* is transformed to solve the two-dimensional optimal solution by Equation (17).

### 3.3. LM Algorithm

The LM known as the damped least-squares (DLS) method, is one of the most effective algorithms to solve nonlinear least squares problems, which has both the advantages of gradient descent method and Gauss-Newton algorithm (GNA). The LM uses a gradient to find the maximum (minimum) value and obtains the optimal solution through iterative convergence. The (non-negative) damping factor *λ* is adjusted at each iteration. When *λ* is used as a smaller value, bringing the algorithm closer to the GNA, whereas if an iteration gives insufficient reduction in the residual, *λ* can be increased, giving a step closer to the gradient-descent direction. We use the LM algorithm to iterate to get the frequency and phase values in the paper. The duration of the coherent integration time is 1.6 ms, which is consistent with the LM iteration time. And the loop adjustment period of the carrier tracking loop is 25 ms. Since the LM iteration time is less than the loop adjustment period, the algorithm proposed in this paper can ensure the real tracking. Therefore, the LM algorithm is used to solve the two-dimensional optimal solution in Equation (17). The iterative optimal solution is given by the following equation:

(18) $${\overset{\wedge }{\mu }}_{i+1}={\overset{\wedge }{\mu }}_{i}+{\left(H_{i}+\lambda \right)}^{-1}G_{i},i=0,1,2\ldots$$

where subscript *i* represents the number of iterations, ${\overset{\wedge }{\mu }}_{i}$ is an 2 × 1 state vector including Δ*f* and Δ*φ*, *λ* is an 2 × 2 diagonal matrix, used to ensure that *H_i_* + *λ* is positive definite and adjust the iterative convergence rate. *H_i_* and *G_i_* is 2 × 2 pseudo-Hessian matrix and 2 × 1 gradient vector respectively. *H_i_* and *G_i_* can be written as:

(19) $$H_{i}={\left[\frac{\partial^{2}M}{\partial \mu^{2}}\right]}_{\mu =\mu_{i}}$$

(20) $$G_{i}={\left[\frac{\partial M}{\partial \mu }\right]}_{\mu =\mu_{i}}$$

where

(21) $$\frac{\partial M}{\partial \mu }={\left[\begin{array}{cc} \frac{\partial M}{\partial \Delta f} & \frac{\partial M}{\partial \Delta \varphi } \end{array}\right]}^{T},\frac{\partial^{2}M}{\partial \mu^{2}}=\left[\begin{array}{cc} \frac{\partial^{2}M}{\partial \Delta f^{2}} & \frac{\partial^{2}M}{\partial \Delta f\partial \Delta \varphi }\\ \frac{\partial^{2}M}{\partial \Delta \varphi \partial \Delta f} & \frac{\partial^{2}M}{\partial \Delta \varphi^{2}} \end{array}\right]$$

**Figure 4:**
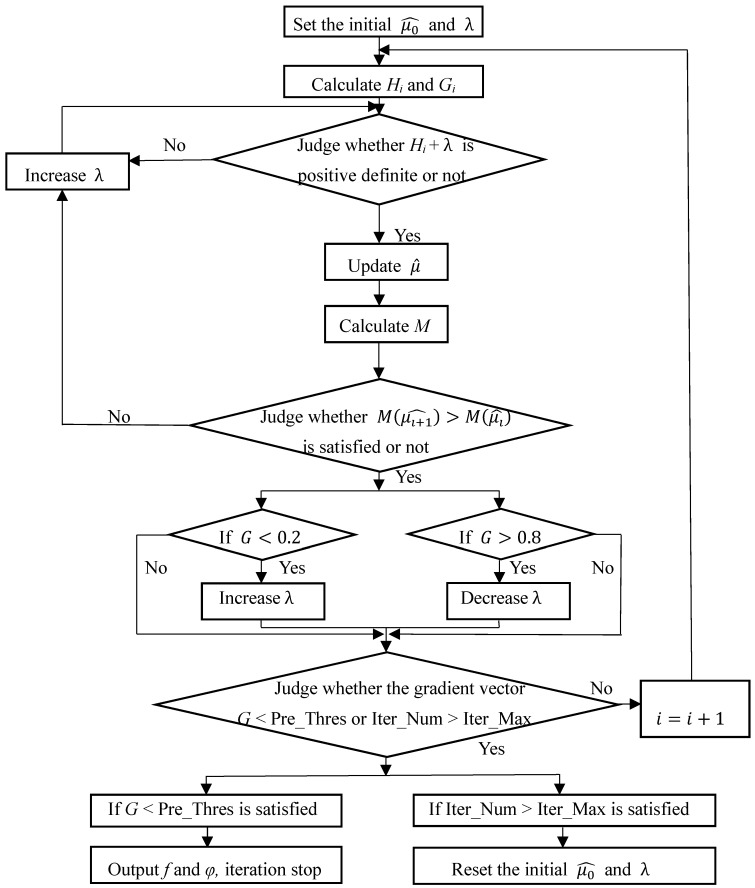
LM algorithm flow chart.

The entire LM algorithm iterative process is shown in Figure 4. The first step is to initialize the value of ${\overset{\wedge }{\mu }}_{0}$ and *λ*. Since we assume that the carrier is correctly tracked at the beginning then ${\overset{\wedge }{\mu }}_{0}=[0\;0]$ and *λ* initialized to an experience value. Next, Equations (19) and (20) are utilized to get *H_i_* and *G_i_*. Increasing *λ* to make *H_i_* + *λ* positive definite. Then Equation (18) is used to update $\overset{\wedge }{\mu }$ and Equation (17) is used to judge whether $M({\overset{\wedge }{\mu }}_{i+1})>M({\overset{\wedge }{\mu }}_{i})$ is satisfied. If not, continue to increase *λ*. If satisfied, judging the gradient vector *G* is less than 0.2 or greater than 0.8 (the judgment condition for *G* is set according to the actual test), then adjust *λ* according to the conditions respectively satisfied. The physical meaning of adjusting *λ* is that if the estimated value $\overset{\wedge }{\mu }$ is closer to the optimal value of iteration, then increase *λ* to slow down the convergence rate, otherwise decrease *λ* to accelerate iterative convergence. The termination condition of the above iterative process is that the value of the gradient vector *G* falls below predefined threshold (Pre_Thres) or the number of iterations (Iter_Num) exceeds the set maximum (Iter_Max).

### 3.4. KF Model

**Figure 5:**
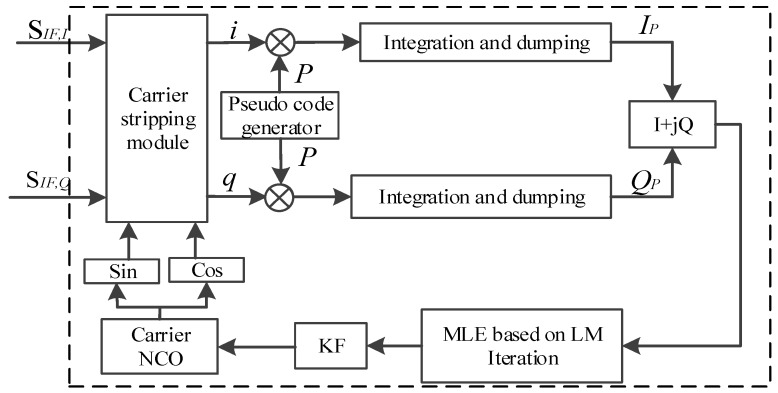
The proposed carrier loop structure based on MLE and KF.

MLE can obtain the minimum error variance of parameters without relying on the prior distribution of signal estimation parameters. For MLE, the choice of the number of observations *N* is significant. The reason is that if a small value of *N* is adopted, the observed sample value of MLE is small, thus the estimated result cannot reflect the true data very well. Conversely, if the observed value of *N* is large, excessive observation noise will be introduced, which is not beneficial to obtaining accurate results. Therefore, if we use the estimated frequency directly in weak signal environment, the non-negligible estimated error in the carrier loop will occur, and finally restrict the improvement of tracking sensitivity and accuracy under weak signals. Since KF is the optimal filter in linear discrete systems [33]. KF is adopted in this paper to smooth the estimation of the parameters after a non-linear estimation by MLE. Therefore, for the carrier frequency tracking problem of TC-OFDM system, the combination algorithm of MLE and KF is the optimal solution. The proposed carrier loop structure is shown in Figure 5.

The main idea is to use MLE to get the estimated value of Δ*f* and Δ*φ*, and KF to smooth the MLE parameter estimation error to get more accurate Δ*f* and Δ*φ*. The output value of the KF is used to adjust the carrier NCO. The MLE uses the LM iteration to get the next value of Δ*f* and Δ*φ*. In practical application, the form of KF is closely related to the state equation and observation equation.

The state equation describes the behaviour of the state vector. In order to adjust the carrier loop accurately, the state vector in the selected carrier loop is expressed as:

(22) $$X=[\begin{array}{ccc} \varphi & \omega_{d} & \omega_{d}^{\prime } \end{array}]$$

where *φ* is the carrier phase estimation value, *ω_d_* = 2*π*Δ*f* denotes the angular frequency estimation value, $\omega_{d}^{\prime }$ represents the angular frequency rate.

The state equation can be expressed as:

(23) $$X_{k}=\Phi X_{k-1}+W_{k-1}$$

where *W_k−_*_1_ is the input Gaussian white noise with mean zero and variance *Q*, Φ is the state transition matrix and the form can be written as:

(24) $$\Phi =\left[\begin{array}{ccc} 1 & T & T^{2}/2\\ 0 & 1 & T\\ 0 & 0 & 1 \end{array}\right]$$

where *T* = *NT_coh_* is the loop update period.

The observation equation describes the relationship between observations and state vectors. The observation equation can be expressed as:

(25) $$Y_{k}=HX_{k}+V_{k}$$

where *H* is the observation matrix, the form can be written as:

(26) $$H=\left[\begin{array}{ccc} 1 & 0 & 0\\ 0 & 1 & 0\\ 0 & 0 & 1 \end{array}\right]$$

The value of the rate of change of Doppler in the observation matrix *H* is a predetermined experienced value. The preset value is reasonable which satisfies the current applications in the TC-OFDM system. And this kind of pre-setting for the rate of change of Doppler is beneficial to us to further study the TC-OFDM receiver algorithm in high dynamic scenario.

*V_k_* is the observed noise, the mean is zero and the variance matrix is *R*. The phase difference Δθ between the input signal and the local signal can be expanded by the Taylor series:

(27) $$\left\{ \begin{array}{l} \Delta \theta (k+1)=\Delta \theta (k)+T\Delta \omega_{0}(k)+\frac{T^{2}}{2}\Delta \omega_{1}(k)+\xi_{1}(k)\\ \Delta \omega_{0}(k+1)=\Delta \omega_{0}(k)+T\Delta \omega_{1}(k)+\xi_{2}(k)\\ \Delta \omega_{1}(k+1)=\Delta \omega_{1}(k)+\xi_{3}(k) \end{array}\right.$$

where Δθ is the carrier phase, $\Delta \omega_{0}$ is the residual carrier and $\Delta \omega_{1}$ is the rate of change of Doppler.

The form of $\xi_{i}$ can be written as:

(28) $$\begin{array}{cc} \xi_{i}(k)=\int_{(k-1)T}^{kT}\frac{\tau^{3-i}}{(3-i)!}Y(\tau )d\tau , & i=1,2,3 \end{array}$$

$\xi_{i}(k)$ is the remainder of Taylor expansion, which is used to represent model noise and describes the effects of random interference and model inaccuracy as described above. Y(t) is a zero-mean, white Gaussian noise process with a single-sided spectral density *N_y_*. The noise variance can be expressed as:

(29) $$E[Y^{2}(t)]=\sigma_{y}^{2}=\frac{N_{y}}{2T}$$

and the value of $E[\xi_{i}(k)\cdot \xi_{j}(k)]$ can be obtained:

(30) $$\begin{array}{l} E[\xi_{i}(k)\cdot \xi_{j}(k)]\\ =E\left\{\left[\int_{(k-1)T}^{kT}\frac{u^{3-i}}{(3-i)!}Y(u)du\right]\left[\int_{(k-1)T}^{kT}\frac{v^{3-i}}{(3-i)!}Y(v)dv\right]\right\}\\ =\iint_{uv}\left\{\frac{u^{3-i}}{(3-i)!}\cdot \frac{v^{3-i}}{(3-i)!}E[Y(u)\cdot Y(v)]\right\}dudv\\ =\frac{\sigma_{y}^{2}\cdot T^{8-(i+j)}}{\left(3-i)!\cdot (3-j)!\cdot [7-(i+j)\right]} \end{array}$$

Then the variance matrix *Q* can be derived:

(31) $$\begin{array}{l} Q=E[\xi (k)\xi^{T}(k)]\\ =E\left\{\left[\begin{array}{c} \xi_{1}(k)\\ \xi_{2}(k)\\ \xi_{3}(k) \end{array}\right]\left[\begin{array}{ccc} \xi_{1}(k) & \xi_{2}(k) & \xi_{3}(k) \end{array}\right]\right\}\\ =\sigma_{w}^{2}I\left[\begin{array}{ccc} T^{4}/20 & T^{3}/8 & T^{2}/6\\ T^{3}/8 & T^{2}/3 & T/2\\ T^{2}/6 & T/2 & 1 \end{array}\right] \end{array}$$

where $\sigma_{w}^{2}=\sigma_{y}^{2}T^{2}$.

The forms of *Q* and *R* can be respectively denoted as [34]:

(32) $$Q=\sigma_{w}^{2}I\left[\begin{array}{ccc} T^{4}/20 & T^{3}/8 & T^{2}/6\\ T^{3}/8 & T^{2}/3 & T/2\\ T^{2}/6 & T/2 & 1 \end{array}\right]$$

(33) $$R=\sigma_{v}^{2}I$$

where *I* is the unit matrix.

The *σ_v_*^2^ in *R* is adjusted according to the value of *A_mp_* estimated by MLE. For the *σ_v_*^2^ in the observation covariance matrix *R*, we obtained a series of experience values based on multiple experiments during the algorithm experiments. According to the estimated value of the signal amplitude *A_mp_* obtained in the MLE, these experience values are utilized to adjust the preset value of the *σ_v_*^2^.

The KF algorithm obtains the filter value at the current time according to the state estimation value at the previous moment and the observation value at the current time. The entire carrier loop filtering process can be divided into two parts, the state estimation and state prediction. The output state estimate *X_k_* of KF and the variance matrix *P_k_* of the estimation error can be iteratively calculated by the following equations:

(34) $$X_{k,k-1}=\Phi X_{k-1}$$

(35) $$P_{k,k-1}=\Phi P_{k-1}\Phi^{T}+Q_{k-1}$$

(36) $$K_{k}=P_{k,k-1}H_{k}^{T}{\left[H_{k}P_{k,k-1}H_{k}^{T}+R_{k}\right]}^{-1}$$

(37) $$X_{kk}=X_{k,k-1}+K_{k}[Y_{k}-HX_{k,k-1}]$$

(38) $$P_{k,k}=[I-K_{k}H]P_{k,k-1}$$

From Equation (36) can be seen that when *R_k_* is large, the corresponding *K_k_* will be small, then the state estimate calculated by Equation (37) is small; when *Q_k_* is small, one step prediction covariance matrix *P_k,k_*_−1_ calculated by Equation (35) will be small, which lead to a smaller state estimate *X_k_*_−1_ finally. From the above analysis, it can be seen that every update of the system state by KF is a compromise between the current system state uncertainty and the observation uncertainty. Therefore, in this paper, *R_k_* and *Q_k_* are obtained by real-time statistics of noise on historical observation and current observation to enhance the adaptability of KF to noise.

## 4. Simulation and Analysis

According to the previous discussion, a novel carrier loop algorithm based on MLE and KF is designed. In order to further illustrate the feasibility and performance of the proposed algorithm, simulations and real data tests are performed in this section. Monte Carlo simulations are adopted to compare the proposed algorithm with the current algorithms to make a comprehensive evaluation of the novel carrier loop algorithm. In addition, the TC-OFDM signals are broadcasted by the modified base stations, a positioning receiver and other related equipment is also utilized to achieve the proposed algorithm. Finally, we choose several points in a test building to test the positioning accuracy for the static receiver.

### 4.1. Simulations

In order to verify the performance of the proposed algorithm, Monte Carlo simulation is applied to evaluate the proposed algorithm effectively and comprehensively. To demonstrate the reliability and effectiveness of the novel carrier loop algorithm, comparative tests are performed. The feasibility of iterative estimation of the LM algorithm, the validity of the KF smoothing error and the superiority of the combination the MLE with the KF algorithm are verified, respectively.

#### 4.1.1. Determine the Number of Observations for MLE

**Figure 6:**
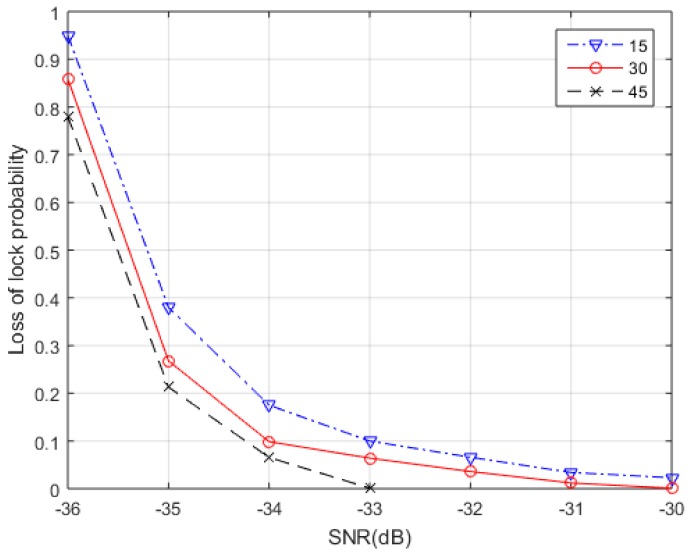
The Relationship between Loss of Lock Probability and SNR.

In statistics, MLE is a method of estimating the parameters of a statistical model. And the estimation accuracy of parameter using MLE is closely related to the number of signal observations used for one estimation. In general, the accuracy of the MLE parameter estimation increases with the number of observations used. Therefore, it is necessary to first analyze how many observations is suitable for one MLE in the carrier loop. The algorithm proposed uses coherent integration results as the MLE observations. We use the loss of lock probability as a standard reference criteria to evaluate the number of observations. The relationship between loss of lock probability and SNR using different observations is shown in Figure 6. The numbers in the legend in Figure 6 represent the values of *N*. The positioning signal adopts Gold codes and the simulation parameters are listed in Table 1.

**Figure 7:**
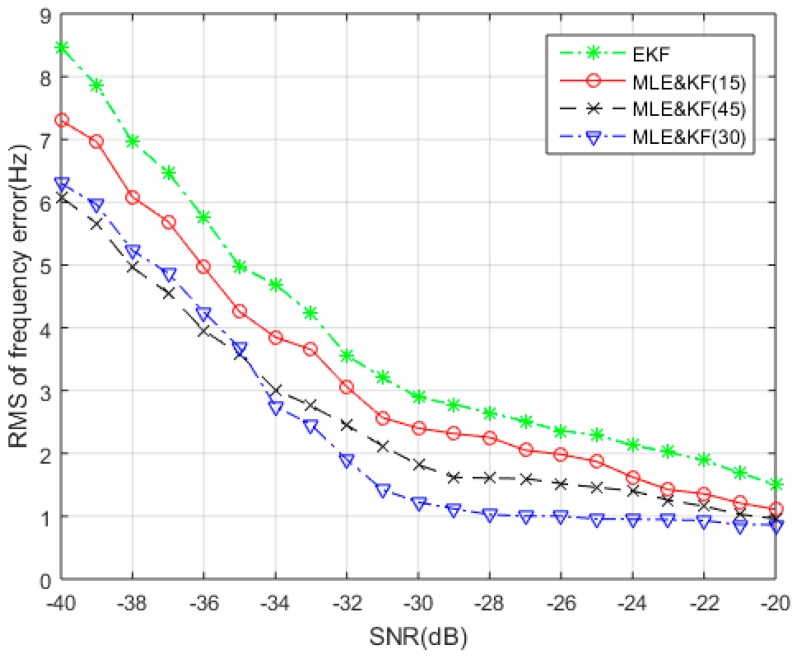
The RMS Frequency Tracking Error with SNR under different sample observations.

Figure 6 shows the correspondence between the loss of lock probability and SNR of the proposed algorithm when *N* = 15, *N* = 30, and *N* = 45. It can be seen from the results that as the number of sample observations (denoted as *N*) gradually increases, the anti-noise ability of the proposed algorithm gradually increases, which means the loss of lock probability with same SNR gradually decreases. The result in Figure 6 is reasonable. Because the value of SNR in experiments is really low, the influence of noise under weak signals will be greater than the different number value of *N*, which is the main factor. We took a piece of data at low SNR to illustrate the problem in TC-OFDM system. Also, 500 Monte Carlo simulations are made to give RMS of frequency error with different sample observations. The results are shown in Figure 7.

Figure 7 gives the RMS frequency tracking error performance curve for the proposed algorithm with different *N* values. The numbers in the legend in Figure 7 represent the values of *N*. From the results, it can be seen that when the SNR is less than −35 dB, the larger of N, the higher frequency estimation accuracy of the MLE&KF. The reason is that the larger number of sample observations leads to the MLE with higher frequency resolution. However, the noise power gradually decreases with the increase of SNR. When the SNR is greater than −35 dB, an excessively large value of *N* will lead to excessive steady state error. Therefore, the error curve corresponding to *N* = 45 will be degraded. In addition, Figure 7 also compares the error performance curve of MLE&KF with EKF. From the comparison results, it can be found that the performance of the frequency tracking error of the MLE&KF designed for the optimal estimation and filtering algorithm is significantly improved than the performance of the EKF based on the sub-optimal filtering algorithm. According to the above analysis, observations with *N* = 30 are used to make a reasonable statistical sample observations.

#### 4.1.2. LM iteration Effectiveness

**Figure 8:**
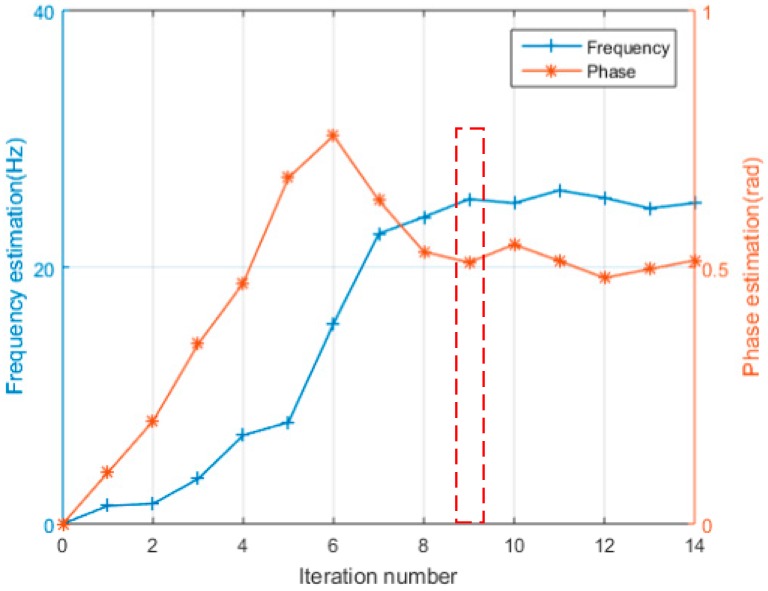
The residual carrier and phase convergence curve estimated by LM algorithm.

To test the feasibility and effectiveness of the MLE and the LM algorithm, we set the residual carrier as 25 Hz and the phase as 0.5 rad of the input TC-OFDM signal. The SNR is −25 dB and other parameters are same as Table 1. The convergence curve of frequency and phase using MLE and iteratively by LM algorithm is shown in Figure 8. From Figure 8, after iteration to the 9th, the residual carrier and phase values remain stable and are close to the set value. The final residual carrier value is stable at 25.8 Hz and the phase value is stable at 0.53 rad, which verifies the feasibility of using MLE to estimate the residual carrier and phase of the carrier loop. For the convenience of analysis, we selected the iteration number fixed at nine in the following tests.

#### 4.1.3. KF Effectiveness of Smoothing Error

**Figure 9:**
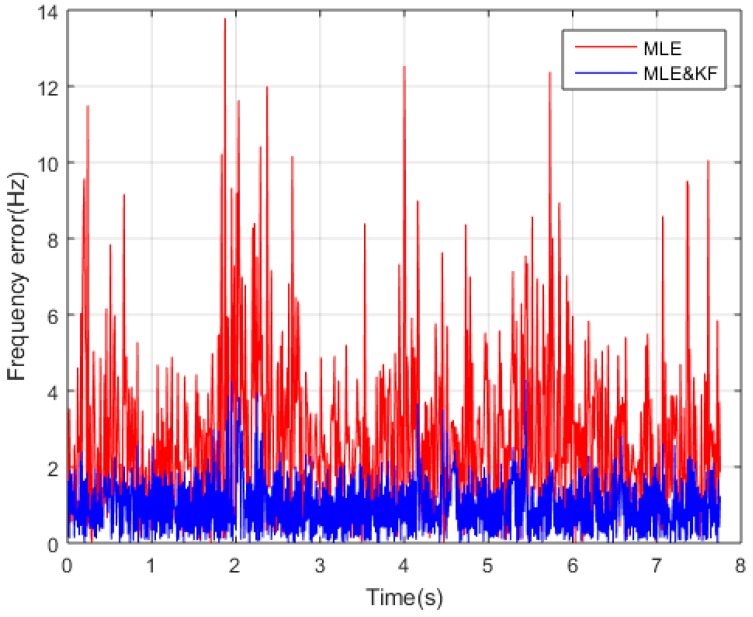
Frequency error comparison results by MLE and MLE&KF.

Meanwhile, there is still a large frequency error in adjusting the carrier loop directly using the signal parameters estimated by MLE for weak TC-OFDM signals. KF is adopted to smoothing the frequency error, so it is necessary to demonstrate the validity of KF smoothing error, and the MLE combined with KF loop is compared with MLE loop. The SNR is −25 dB and other parameters are same as Table 1. Figure 9 shows that the error of frequency estimation after KF is obviously decreased in contrast to using MLE only, which verified the validity of KF smooth frequency error. The MLE combined with KE denotes as MLE&KF and the other denotes as MLE in Figure 9, respectively.

#### 4.1.4. Performance Comparison Results of the Proposed Algorithm and Other Algorithms

**Figure 10:**
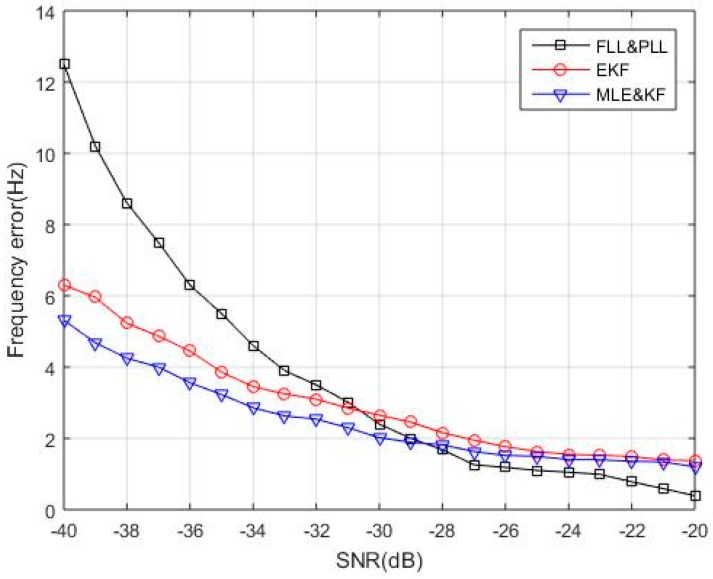
Comparison results of frequency estimation errors by three algorithms under different SNR.

To verify the signal parameter estimation accuracy corresponding to different SNR, we compare the proposed algorithm with the conventional second-order FLL assisted third-order PLL (FLL&PLL) carrier loop and EKF algorithm. The FLL&PLL displays both the high accuracy of PLL and the large dynamics of FLL. The specific parameters and corresponding discriminator algorithm of the second-order FLL assisted third-order PLL are given in Table 2. The EKF algorithm used for comparison in this paper is a pure EKF algorithm that is independent of MLE. The result of the coherent integration is used as the input observation signal parameter of the EKF. Since the carrier loop can be considered as a nonlinear discrete-time system. The purpose of using the EKF-based carrier loop is to linearize the state equation and the observation equation of carrier tracking loop to obtain a sub-optimal estimation method for the carrier loop. The algorithm proposed in this paper is to use MLE to estimate the interested parameters of the carrier loop, and then use KF to filter estimated parameters. Since the carrier loop can be considered as a nonlinear discrete-time system. The KF is a linear discrete system filter which cannot be directly used to the carrier loop. We use MLE to estimate the carrier loop parameters and do a non-linear approximation, and then use KF for filtering, which is different from the pure EKF algorithm. Two hundred Monte Carlo simulations are performed for each SNR with a fixed predetermined frequency error. The RMSE frequency error comparison results are shown in Figure 10. The results in Figure 10 show that the frequency estimation errors of the three algorithms all increase with SNR decreasing. Among them, the FLL&PLL has the largest error under low SNR, followed by the EKF. The proposed algorithm (MLE&KF) can still effectively estimate the frequency, which shows high frequency estimation accuracy and tracking performance for weak TC-OFDM signals. The simulation verifies the superiority of the proposed algorithm for various SNR.

**Figure 11:**
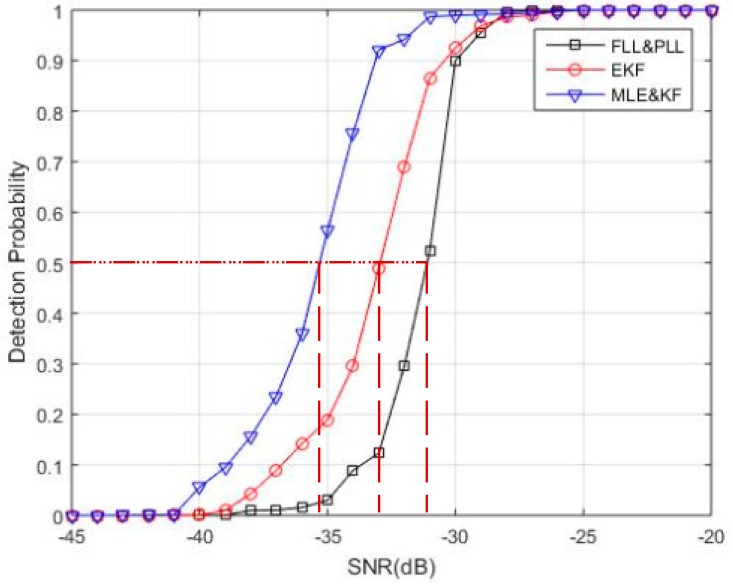
Comparison of the tracking probabilities between the three algorithms.

Meanwhile, in order to further verify the tracking performance of the proposed algorithm, the comparison results of the tracking probability with other two algorithms are shown in Figure 11. Tracking probability is defined as the probability of successfully tracking the total number of Monte Carlo simulations. Tracking sensitivity is defined as the SNR at which tracking probability exceeds 50%. At present, the definition of tracking sensitivity can be divided into two types: the detection probability is 50% [31] and 90% [35]. In order to better illustrate the problem, this paper refers to [31] for the definition of tracking sensitivity. According to the results of the detection probability of the proposed algorithm in Figure 11, the SNR is adopted to be taken as the tracking sensitivity when the tracking probability is 50%, which can reflect the overall level of the performance of the algorithms. It can be seen that the tracking sensitivity of FLL&PLL is −31.1 dB, followed next by EKF which is −33 dB. The MLE&KF algorithm has better performance than the others, and the tracking sensitivity is −35.3 dB, which is 2.3 dB lower than EKF and 4.2 dB lower than FLL&PLL. The Monte Carlo simulation results prove that proposed algorithm can significantly improve the tracking sensitivity for weak TC-OFDM signals.

### 4.2. Real Data Tests

**Figure 12:**
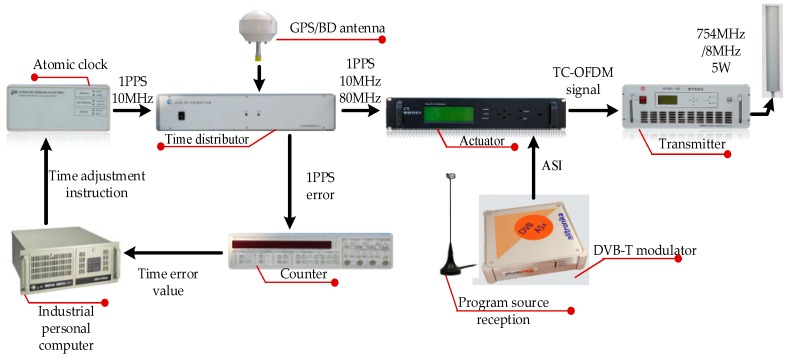
Each component of the modified base stations.

**Figure 13:**
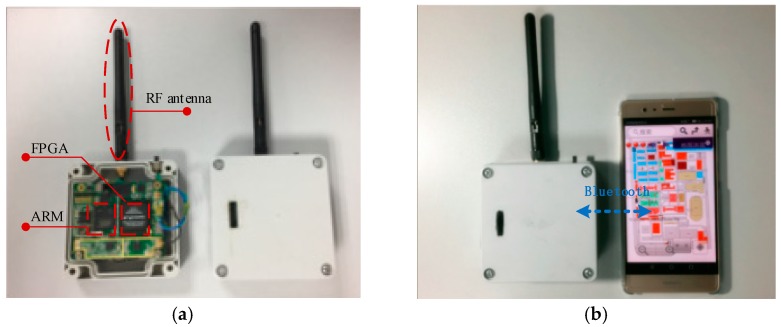
The TC-OFDM receiver. (**a**) is the internal and external structure of the TC-OFDM receiver; and (**b**) is the communication between the positioning receiver and the mobile phone.

Real data tests are conducted to prove that the novel carrier loop algorithm has significantly good performance in an actual environment. We start our actual tests in a comprehensive test platform, which is shown in Figure 12 and Figure 13. The test platform consists of a modified base station and the TC-OFDM receiver. Figure 12 describes each component of the modified base station in detail. The time distributor, the counter and the industrial personal computer collaborative work together in order to ensure synchronization between the modified base stations. Each modified base station equipped with an atomic clock with an output frequency of 10 MHz. The synchronization accuracy between the modified base stations is up to 5 ns (1σ). Meanwhile, the time distributor generates the positioning data messages containing UTC, air pressure, base station number and base station coordinates which are necessary for positioning. The function of the actuator is to modulate the TC-OFDM signal into RF signal and finally the RF signal is transmitted by the transmitter.

We use the TC-OFDM receiver developed by us which is shown in Figure 13 to test the positioning accuracy. An overview of the internal and external structures of the TC-OFDM receiver is shown in Figure 13a. Figure 13b shows that the TC-OFDM receiver and mobile phone communicate through the Bluetooth protocol to transmit useful data messages. Then the phone exhibits the final positioning result via the map. An architecture based on FPGA and ARM is adopted in the TC-OFDM receiver for baseband processing and data demodulation. The FPGA is used to perform the main logic operations and ARM is responsible for controlling the circuit logic. The IF signal is processed into a zero-digital IF signal for subsequent FPGA processing. The LM iterative and KF algorithm used in the proposed carrier loop are implemented in the ARM processer. The main computational load is in LM. In each LM iteration, sine, cosine operations and matrix calculations are needed. The above calculations are easily calculated in ARM which can meet real tracking requirements for carrier loop.

**Figure 14:**
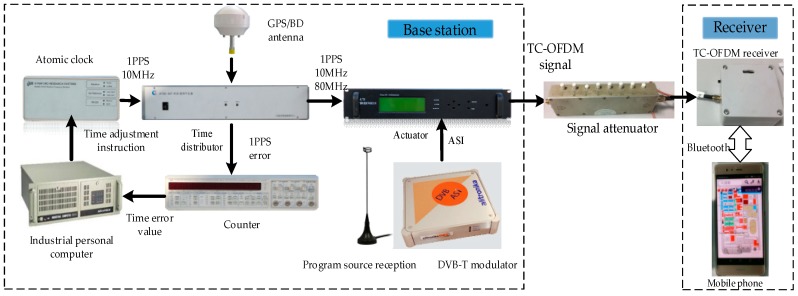
Actual test diagram of the tracking sensitivity between the three algorithms.

In addition, we implement the proposed algorithm in our own developed TC-OFDM receiver. The EKF algorithm and two-order FLL assisted three-order PLL algorithm are also implemented to make a comparison. The performance when the three algorithms track weak TC-OFDM signals can be evaluated in practical applications as shown in Figure 14. The modified base station is utilized to generate TC-OFDM signals. Then the TC-OFDM signal is sent to a signal attenuator. The signal attenuator is used to set different signal power. Then the receiver receives TC-OFDM signal of different signal power for subsequent baseband processing. Finally, the useful data is transmitted to the mobile phone for positioning. We use signal attenuator to gradually reduce the signal power to test the ability of tracking weak signal performance of three algorithms. The test results are shown in Table 3, where it can be seen that the proposed algorithm has better tracking sensitivity for weak TC-OFDM signals by 2–4 dB. In the real TC-OFDM system, the weak signal power is generally between −125 dBm to −130 dBm. But, under the special cases, the signal power is low to −136 dBm. Therefore, the sensitivity improvement of the proposed algorithm is modest, and it can satisfy the use of existing TC-OFDM systems. Meanwhile, the proposed carrier loop tracking algorithm in this paper is implemented in the receiver’s ARM structure. The ARM development resources of existing TC-OFDM receivers can meet the complexity and computational burden of the proposed algorithm. Furthermore, from Table 3, the proposed algorithm can effectively track the TC-OFDM signal when the signal power is −136 dBm. The signal power using the algorithm of FLL-assisted PLL and pure EKF is −132 dBm and −134 dBm, respectively. For signals with lower signal power, these two algorithms can no longer track steadily, and even lose lock, so the improvement of signal tracking sensitivity of the proposed algorithm is significant for practical applications.

**Figure 15:**
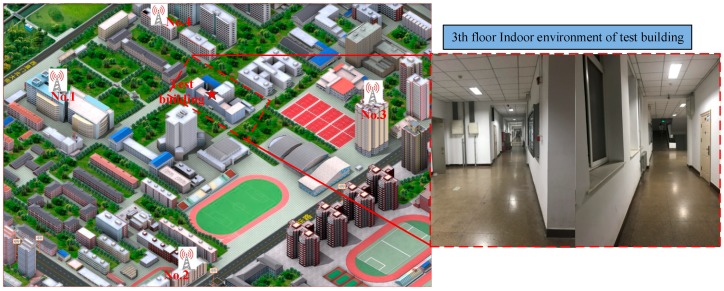
The base station distribution of the test environment on the campus.

**Figure 16:**
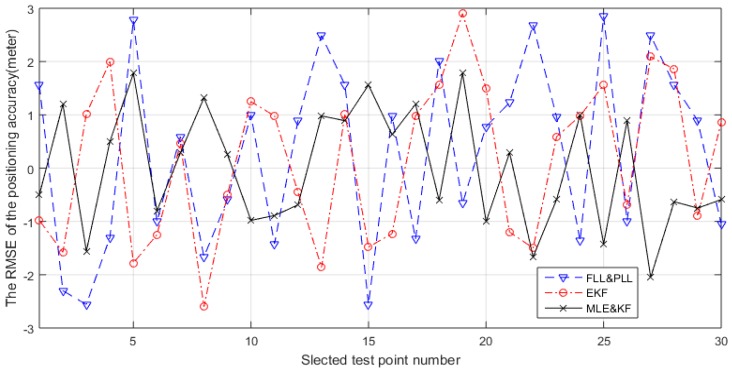
The RMSE positioning accuracy error in horizontal direction.

Furthermore, we ran an actual test on the campus suing the test environment shown in Figure 15. We set up the base station on the roof of four buildings on our campus. Then we selected the 3th floor of another building as test building to start our real test using the TC-OFDM receiver. The two-order FLL assisted three-order PLL and EKF algorithms are implemented in the receiver to compare with the proposed algorithm. In order to verify the better performance of the proposed algorithm for weak TC-OFDM signal, we selected 30 test points on the floor of the test building for positioning accuracy test. The receiver is placed at each test point for ten minutes, then we calculated the Root-mean-square error (RMSE) of the test points. The corresponding acquisition and positioning algorithms are utilized for the horizontal positioning. Since the positioning system uses a custom coordinate system for indoor positioning, the output positioning result is compared with the distance between the selected point and the origin of the corresponding floor. The RMSE of the positioning accuracy error results of the three algorithms between the selected point and the corresponding original point are shown in Figure 16, which shows the horizontal measurement accuracy results of three algorithms. The horizontal axis in the figure represents the sequence number corresponding to the selected 30 points. The vertical axis represents the calculation error of the final positioning result corresponding to each selected point. The positioning results of the blue, red, and black lines respectively represent the three different carrier loop algorithms: FLL&PLL, EKF, and MLE&KF. The final positioning results of using MLE&KF has the smallest fluctuations and the best positioning accuracy, which shows the superior performance of our algorithm compared to other algorithms. Also, these results is verified the effectiveness of the proposed algorithm in practical applications. The horizontal positioning accuracy is better than 3 m. The TC-OFDM receiver can be used to achieve accurate positioning in many indoor LBS applications, such as indoor mall pedestrian navigation and positioning and elderly care terminal. It can be seen that the positioning accuracy obtained by the proposed algorithm has smaller positioning error and higher positioning accuracy than the others for weak TC-OFDM signals.

## 5. Conclusions

To improve the tracking performance for weak TC-OFDM signals, a novel carrier loop algorithm based on MLE and KF is proposed in this paper. The algorithm derives the MLE discriminator function to replace the existing discriminator in carrier loop. The Levenberg-Marquardt (LM) algorithm is utilized to obtain the MLE cost function consisting of signal amplitude, residual carrier frequency and carrier phase, and the MLE discriminator function is derived from the corresponding MLE cost function. In order to further reduce the residual frequency error, the KF is used to smooth the MLE discriminator function results. Numerical simulation and real data tests are implemented to verify the performance of the algorithm. The test results indicate that the novel carrier loop algorithm can improve the tracking sensitivity of the TC-OFDM receiver by taking full advantage of the characteristics of the carrier loop parameters. Finally, compared with the current carrier loop algorithm, the tracking sensitivity is effectively improved by 2–4 dB and the positioning accuracy is experimentally improved in the real environments.

## Figures and Tables

**Table 1:** Simulation parameters.

Parameter	Value
Slot time, *T_F_*	25 ms
Sample rate	4.4
Intermediate frequency, *f_IF_*	0 Hz
Data bit transition	Random
Coherent integration time, T*_coh_*	1.6 ms
Predefined threshold, Pre_Thres	0.02
Iteration maximum numbers, Iter_Max	15
Signal to noise ratio, SNR	−45 dB–−20 dB

**Table 2:** The specific parameters and corresponding discriminator algorithms of second-order FLL assisted third-order PLL.

Parameter	Value
PLL bandwidth, *B_w,PLL_*	10 Hz
FLL bandwidth, *B_w,FLL_*	20 Hz
*Wn* of carrier PLL	*B_w,PLL_*/0.53
*Wn* of carrier FLL	*B_w,FLL_*/0.53
Phase discrimination equation	$Q_{p}\cdot sign(I_{p})/sqrt(I_{p}{}^{2}+Q_{p}{}^{2})$
Frequency discrimination equation	$P_{cross}\cdot sign(P_{dot})/\left(I_{p}{}^{2}+Q_{p}{}^{2}\right)$ where $P_{cross}=I_{p-1}\cdot I_{p}+Q_{p-1}\cdot Q_{p}$, $P_{dot}=I_{p-1}\cdot Q_{p}-Q_{p-1}\cdot I_{p}$

**Table 3:** Tracking sensitivity of three algorithms under different signal powers.

Signal Power (dBm)	−126	−127	−128	−129	−130	−131	−132	−133	−134	−135	−136
the proposed algorithm	O ^1^	O	O	O	O	O	O	O	O	O	O
EKF	O	O	O	O	O	O	O	O	O	-	-
FLL&PLL	O	O	O	O	O	O	O	- ^2^	-	-	-

^1^ The mark O represents the algorithm can track the signal at the corresponding signal power. ^2^ The mark—represents the algorithm cannot track the signal at the corresponding signal power.

## References

[1] Shen Y., Win M.Z. (2010). Fundamental Limits of Wideband Localization—Part I: A General Framework. IEEE Trans. Inf. Theory.

[2] Shen Y., Wymeersch H., Win M.Z. (2010). Fundamental Limits of Wideband Localization—Part II: Cooperative Networks. IEEE Trans. Inf. Theory.

[3] Witrisal K., Meissner P., Leitinger E., Shen Y., Gustafson C., Tufvesson F., Haneda K., Dardari D., Molisch A.F., Cont A. (2016). High-Accuracy Localization for Assisted Living: 5G systems will turn multipath channels from foe to friend. IEEE Signal Process. Mag..

[4] Bartoletti S., Dai W., Conti A., Win M.Z. (2015). A Mathematical Model for Wideband Ranging. IEEE J. Sel. Top. Signal Process..

[5] Mazuelas S., Conti A., Allen J.C., Win M.Z. (2018). Soft Range Information for Network Localization. IEEE Trans. Signal Process..

[6] Cecchini G., Bazzi A., Masini B.M., Zanella A. Localization-based resource selection schemes for network-controlled LTE-V2V. Proceedings of the 2017 International Symposium on Wireless Communication Systems (ISWCS).

[7] Bazzi A., Masini B.M., Zanella A., Thibault I. (2017). On the Performance of IEEE 802.11p and LTE-V2V for the Cooperative Awareness of Connected Vehicles. IEEE Trans. Veh. Technol..

[8] Deng Z., Yu Y., Yuan X., Wan N., Yang L. (2013). Situation and Development Tendency of Indoor Positioning. China Commun..

[9] Liu W., Xi Y., Deng Z., Jiao J., Yin L. (2015). Correlation combination ambiguity removing technology for acquisition of sine-phased BOC(kn,n) signals. China Commun..

[10] Deng Z., Mo J., Jia B., Bian X. (2017). An Acquisition Scheme Based on a Matched Filter for Novel Communication and Navigation Fusion Signals. Sensors.

[11] Chiurco G., Mazzotti M., Zabini F., Dardari D., Andrisano O. (2012). FPGA Design and Performance Evaluation of a Pulse-Based Echo Canceller for DVB-T/H. IEEE Trans. Broadcast..

[12] Zabini F., Mazzotti M., Dardari D., Chiurco G., Andrisano O. (2014). Performance and Stability Analysis of Echo Cancellers Based on Training Sequences. IEEE Trans. Broadcast..

[13] Zabini F., Pasolini G., Andrisano O. (2016). Design Criteria for FIR-Based Echo Cancellers. IEEE Trans. Broadcast..

[14] Kaplan E.D., Hegarty C. (2006). Understanding GPS: Principles and Applications.

[15] Jin T., Yuan H., Zhao N., Qin H., Sun K., Ji Y. (2017). Frequency-Locked Detector Threshold Setting Criteria Based on Mean-Time-To-Lose-Lock (MTLL) for GPS Receivers. Sensors.

[16] Lopez-Salcedo J., Peral-Rosado J., Granados G.S. (2014). Survey on robust carrier tracking techniques. IEEE Commun. Surv. Tutor..

[17] Curran J.T., Lachapelle G., Murphy C.C. (2012). Improving the design of frequency lock loops for GNSS receivers. IEEE Trans. Aerosp. Electron. Syst..

[18] Xie G. (2009). Principles of GPS and Receiver Design.

[19] Vilà-Valls J., Closas P., Fernández-Prades C. On the identifiability of noise statistics and adaptive KF design for robust GNSS carrier tracking. Proceedings of the 2015 IEEE Aerospace Conference.

[20] Gandhi M.A., Mili L. (2010). Robust Kalman filter based on a generalized Maximum–Likelihood–type estimator. IEEE Trans. Signal Process..

[21] Duan R., Liu R., Zhou Y., Song Q., Li Z. (2013). A Carrier Acquisition and Tracking Algorithm for High-Dynamic Weak Signal. Proceedings of the 26th Conference of Spacecraft TT&C Technology.

[22] Zeng C., Li W. Application of Extended Kalman Filter for tracking high dynamic GPS signal. Proceedings of the IEEE International Conference on Signal and Image Processing.

[23] Sun P., Tang X., Huang Y., Chen H., Sun G. (2016). Adaptive wavelet denoising unscented Kalman filter for BeiDou signal carrier tracking under ionospheric scintillation conditions. J. Appl. Remote Sens..

[24] Ward P.W. Performance comparison between FLL, PLL and a novel FLL-assisted PLL carrier tracking loop under RF interference conditions. Proceedings of the 11th International Technical Meeting of the Satellite Division of The Institute of Navigation (ION GPS 1998).

[25] Hurd W.J., Statman J.I., Vilnrotter V.A. (1987). High Dynamic GPS Receiver Using Maximum Likelihood Estimationand Frequency Tracking. IEEE Trans. Aerosp. Electron. Syst..

[26] Jong-Hoon W., Pany T., Eissfeller B. (2012). Iterative Maximum Likelihood Estimators for High-Dynamic GNSS Signal Tracking. IEEE Trans. Aerosp. Electron. Syst..

[27] Won J.H., Pany T., Eissfeller B. (2012). Noniterative Filter-Based Maximum Likelihood Estimators for GNSS Signal Tracking. IEEE Trans. Aerosp. Electron. Syst..

[28] Closas P., Fernandez-Prades C., Fernandez-Rubio J.A. (2007). Maximum likelihood estimation of position in GNSS. IEEE Signal Process. Lett..

[29] Vilnrotter V., Hinedi S., Kumar R. (1989). Frequency estimation techniques for high dynamic trajectories. IEEE Trans. Aerosp. Electron. Syst..

[30] Trees H.L.V. (1968). Detection, Estimation, and Modulation Theory. Part III. Papoulis Probab. Random Var. Stoch. Process..

[31] Zhang H., Yan B., Zhang H., Luo L. (2017). A Carrier Estimation Method Based on MLE and KF for Weak GNSS Signals. Sensors.

[32] Borio D., Lachapelle G. (2009). A non-coherent architecture for GNSS digital tracking loops. Ann. Telecommun..

[33] He Z., Petovello M. Performance comparison of Kalman filter and maximum likelihood carrier phase tracking for weak GNSS signals. Proceedings of the 2015 International Conference on Indoor Positioning and Indoor Navigation.

[34] Wu W., Chen H., Ting Z., Cui W. High dynamic carrier tracking technology based on Kalman filter. Proceedings of the IET International Radar Conference.

[35] Tangudu J., Ramasubramanian K., Subburaj K., Khanna S., Chomal S. Techniques to enhance GNSS signal acquisition and tracking sensitivity. Proceedings of the International Conference on Indoor Positioning and Indoor Navigation.

